# Variations in structural and physicochemical properties of lotus seed starch–protein blends under various HHP treatment conditions

**DOI:** 10.1016/j.fochx.2025.102281

**Published:** 2025-02-12

**Authors:** Sidi Liu, Ru Jia, Wenjing Chen, Wenyu Chen, Baodong Zheng, Zebin Guo

**Affiliations:** a College of Food Science, Fujian Agriculture and Forestry University, Fuzhou 350002, China; b College of Food Science and Nutritional Engineering, China Agricultural University, Beijing 100083, China; c Fujian Provincial Key Laboratory of Quality Science and Processing Technology in Special Starch, Fujian Agriculture and Forestry University, Fuzhou 350002, China

**Keywords:** Lotus seed, Seed starch and protein, High hydrostatic pressure, Blends, Structure, Physicochemical properties

## Abstract

The interaction between starch and proteins is a common phenomenon in food processing, which considerably influences food quality. This study investigated the effect of different pressure levels (0.1–600 MPa, 10 min) and holding times (400 MPa, 10–60 min) under high hydrostatic pressure treatment parameters on structures and physicochemical properties of lotus seed starch–protein (LS–LP) blends. Subsequent examination by Fourier transforms infrared spectroscopy and UV–visible absorption spectra revealed stronger interaction between LS and LP with a change in the hydrogen bond content. Scanning Electron Microscope results showed that LS and LP existed in a blended form. X-ray diffraction revealed that the crystallinity decreased with an increase in treatment intensity of LS–LP blends. The improved water absorption capacity of LS–LP blends (<400 MPa) enhanced viscosity, swelling, and solubility power. This study presents a novel practical method of preparing LS–LP blends and provides insights into physicochemical properties to facilitate processing of LS–based food.

## Highlights

• The relationship between high hydrostatic pressure (HHP) and changes of LS-LP blends properties was explored.
• The optimal the HHP conditions for preparation of LS-LP blends were selected.
• High-water absorption capacity of the LS-LP blends were improved by HHP treatment.

## 1 Introduction

Starch and protein are important food components of human diet, with a high likelihood of interaction (Qin et al., 2023). Starch–protein interactions affect the physicochemical properties of foods, including rheological, gelatinization, and thermal properties, which could further influence the final quality, texture, and flavor of food during food processing (Alvarez, Ramaswamy, & Ismail, 2008). Several studies have investigated interactions between starch and proteins. For example, the storage modulus of mixed gels primarily stems from the interactions between cassava starch and whey protein isolate (Ren & Wang, 2019). Gelatinization temperature of wheat starch is reduced by adding gliadin and glutenin (Li et al., 2020). Rice protein has been shown to inhibit the retrogradation of rice starch (Zhang, Chen, Chen, & Chen, 2019). Understanding the mechanisms and physicochemical properties of these interactions is crucial for enhancing the quality of final food products.

Thermal processing is a fundamental method in food processing under elevated temperatures that facilitating interactions among food compounds, particularly interactions between starch and proteins, leading to the establishment of starch–protein blended systems. Starch is gelatinized under a high temperature, which leaches out more amylose and amylopectin molecules to promote interaction with heated proteins with fully unfolded structures (Gui et al., 2022). This method is advantageous for its ease of operation and control. However, high temperatures severely damage starch and protein structures, thereby distorting the original starch particle morphology and causing Maillard reaction, which affect the original food flavor (BeMiller & Huber, 2015). Nonthermal modification treatments are strongly recommended in conjunction with gentler processing methods to reduce the adverse effects of heat processing (Baboli, Williams, & Chen, 2020).

Unlike conventional thermal treatment methods, high hydrostatic pressure (HHP) treatment is a nonthermal technique, which utilizes pressure exceeding 100 MPa to induce modifications in the structural and functional attributes of food. This approach maintains ambient temperature conditions, thereby mitigating the Maillard reaction-induced flavor changes and ensuring maximum retention of nutrient content, organoleptic qualities, and functional properties inherent to the food matrix (Haghayegh & Schoenlechner, 2011). The unique processing attributes of HHP treatment allow starch and proteins to maintain their granular morphology and structural integrity, which leads to the creation of starch–protein blends that demonstrates interaction modalities distinct from those observed in thermally processed equivalents (Pei, Hu, & Shen, 2010). Such differential interactions influence the structure and physicochemical characteristics of starch–protein blends, ultimately affecting the quality of food products (Guo et al., 2015; Su et al., 2022). Therefore, elucidating the variations in structural and physicochemical properties of starch-protein blends under various HHP treatment conditions is essential for improving the quality of starch-protein-based foods. However, research on how HHP treatment and related parameters affect the physicochemical properties of starch–protein blends is limited.

Our previous studies have explored the interaction of high amylose lotus seed starch with other substances under high hydrostatic pressure (HHP) treatment, including lotus seed starch–conjugated linoleic acid and amylose-long-chain fatty acid complexes, which exhibited stable structures and improved physicochemical properties (Jia, Sun, Chen, Zheng, & Guo, 2018; Liu et al., 2022). However, previous studies on lotus seed have not adequately addressed the interactions between starch (50 % dry basis) and other primary components, including protein (20 % dry basis) under HHP treatment, limits the utilization value of lotus seeds in processed foods (Liu et al., 2022). Therefore, the objective of this study was to: (1) investigate the effect of HHP treatment on the structure and physicochemical properties of lotus seed starch–protein blends under different HHP treatment intensities, (2) elucidate the mechanisms underlying the lotus seed starch–protein blends by performing structural analysis at different scales (particle, aggregated, and short-range structures), and (3) determine the thermal and gelatinization properties of lotus seed starch–protein blends. The outcomes of this study are expected to lay a theoretical foundation for the development of starch–protein blends using nonthermal modification technology and enhancing the industrial applications of lotus seed starch and protein.

## 2 Materials and methods

### 2.1 Materials

Fresh lotus seeds were sourced from the Green Field Food Co. Ltd. (Fujian, China) and chemicals used in this study, which were of analytical grade, were purchased from Sinopharm Chemical Reagent Co. Ltd. (Shanghai, China).

#### 2.1.1 Extraction of Lotus seed starch (LS)

Lotus seed starch was isolated as described previously (Guo et al., 2015), with minor modifications. Fresh lotus seeds were soaked in distilled water (30 min), pulverized using a grinder (Xiangtian Experimental Instrument Factory Co., Ltd., Changzhou, China), filtered through a 100-mesh sieve, and the resulting suspension was allowed to stand at ambient temperature for 6 h. The precipitate that formed was washed three times with distilled water and three times with 95 % alcohol, then dried in an oven (Yiheng Technology Co., Ltd., Shanghai, China) overnight at 45 °C. Afterward, the precipitate was pulverized and passed through a 100-mesh sieve to obtain lotus seed starch powder. Lotus seed starch contained, on average, 9.32 % (d.b.) moisture, 0.24 % (d.b.) ash, 0.33 % (d.b.) protein and 0.17 % (d.b.) lipid.

#### 2.1.2 Extraction of Lotus seed protein (LP)

Lotus seed protein was isolated as described previously (Su et al., 2022)^,^ with minor modifications. Dried lotus seeds were pulverized in a grinder and the resulting powder (500 g) was added to distilled water (5 L), adjusted to pH of 11.0 with 0.5 mol L^−1^ NaOH, stirred for 3 h at room temperature, and stored at 4 °C for 24 h. The supernatant was collected after centrifugation at 4500 ×*g* for 10 min. Thereafter, the pH was adjusted to 4.8 by adding 0.1 mol L^−1^ HCl solution and the solution was stirred for 3 h at room temperature and stored at 4 °C for 24 h. The precipitated protein was collected by centrifugation at 4500 ×*g* for 10 min, redissol*v*ed in deionized water (1:10, *w*/*v*) at a neutral pH, and lyophilized in a freeze dryer (FDU-1200 Rikakikai Co., Ltd., Tokyo, Japan). Finally, lotus seed protein powder was obtained. (The lotus seed protein content was determined by the Kjeldahl method with 90.42 %).

### 2.2 Preparation of Lotus seed starch–protein blends (LS–LP)

Lotus seed starch was dispersed in distilled water (6 %, *w*/*v*) and then mixed with LP solutions (3 %, w/v). The pH of the blends was maintained at 7.4 by adding a phosphate buffer and dispersed evenly. The blends were then placed in polypropylene vacuum bags and sealed using a vacuum packaging machine. The samples were stirred thoroughly and transferred into HHP treatment equipment. HHP processing was performed at 5 L-HPP-600 MPa (JiuJiu High Pressure Technology Co., Ltd., Baotou, China) at different predefined pressure levels (0, 100, 200, 300, 400, and 500 MPa) and holding times (0, 10, 20, 30, 40, 50, and 60 min) at room temperature. All treated samples were stored at 4 °C for 48 h, then freeze–dried, ground into powder, and passed through a 100-mesh sieve. The resulting lotus seed starch–protein blend powders were stored in a desiccator until use.

### 2.3 Fourier transform infrared (FTIR) spectroscopy

The FTIR spectra were recorded on an FTIR spectrometer (Bruker, Karlsruhe, Germany) with slightly modification (Qin et al., 2025). The samples (10 mg) and potassium bromide (500 mg) were dried for 12 h at 105 °C, thoroughly mixed, and made into thin disks for spectrometry. The wavenumber range was 4000–400 cm^−1^ and a resolution of 4 cm^−1^.

### 2.4 UV–visible (UV–vis) spectroscopy

Spectra for standard barium sulfate (BaSO_4_) sample cell were recorded on a spectrophotometer (UV-3600i Plus; Shimadzu, Kyoto, Japan) at a wavelength range of 200–400 nm and a 1.0 cm path length as a baseline. Subsequently, standard BaSO_4_ sample cell was removed from the integrating sphere and replaced with a sample cell pressed with all samples.

### 2.5 X-ray diffraction (XRD)

XRD analysis of the starch granules was performed using an X-ray diffractometer (Rigaku Corporation, Tokyo, Japan), as described previously (Conde, Kebede, Leong, & Oey, 2022) with a few modifications. The scan range was from 5° to 35° (2θ), 40 kV, 200 mA, and Cu-Kα radiation. The relative crystallinity was calculated as the area ratio of the crystalline peak over the total area using PeakFit 4.0 (Beijing, China).

### 2.6 Scanning Electron microscopy (SEM)

The surface morphology of granular samples was observed using a scanning electron microscope (Nova NanoSEM 230; FEI, Hillsboro, OR, USA). Samples were attached to the operating platform with a double-sided conductive adhesive tape and covered with a thin layer of gold. The surface morphology was examined at an accelerating voltage of 5 keV.

### 2.7 Evaluation of pasting properties

Pasting profiles of the samples were determined using a Rapid Visco Analyzer (RVA; Anton Paar GmbH, Graz, Austria). The LS–LP blends (2.0 g) were accurately weighed and suspended in deionized water (25 mL) to a final concentration of 8 %. The samples were weighed on the dry basis of native lotus seed starch in an aluminum RVA sample canister. A programmed heating and cooling cycle were used, where the samples were pasted at an initial temperature of 50 °C for 1 min, heated to 95 °C at 5 °C/min, held for 10 min, cooled to 50 °C at 5 °C/min, and held for 2 min.

### 2.8 Evaluation of thermal properties

The pasting thermal parameters of the samples were evaluated using a differential scanning calorimeter (DSC 214 Polyma; Netzsch, Selb, Germany), according to a previously described method (Zhao et al., 2024) with minor modifications. Distilled water (10 μL) was added to the samples (3 g) in a sealed crucible and left to stand at ambient temperature for 12 h. An empty crucible was used as the reference. To take measurements, the temperature was increased from 25 °C to 150 °C at a rate of 10 °C/min.

### 2.9 Analysis of swelling power and solubility of LS–LP blends

Distilled water was added to the emulsion (2 %, LS–LP blends, [1 g]) and oscillated in a water bath at different temperatures (25, 65, and 95 °C) for 30 min. The dried aluminum boxes, which contained supernatant from the centrifuge tubes (5000 ×*g* for 10 min), were dried to a constant weight and the weight gain of the aluminum boxes was recorded as the mass of dissolved starch (A). The mass of the precipitate in the centrifuge tubes was recorded as the mass of starch that had swollen (P). We calculated the swelling power (B) and solubility (S) of the LS–LP blends according to the following equations:

(1) $$\;S\left(\%\right)=\frac{A}{W}\times 100$$

(2) $$\;B\left(\%\right)=\frac{P}{W\left(1-S\right)}\times 100$$

### 2.10 Data analysis

All experiments were performed in triplicate. All images were processed using Origin software (OriginPro 2018C; MicroCal Inc., Northampton, MA, USA) transformation based on the experimental data. Data were analyzed using one-way analysis of variance, which was performed using IBM SPSS Statistics 23.0 (IBM Corp., Armonk, NY, USA). Different test was evaluated by using Duncan Multiple Comparison testing. The significance level was set at *p* < 0.05. Data are expressed as means ± standard deviations.

## 3 Results and discussion

### 3.1 FTIR spectroscopy

**Fig. 1:**
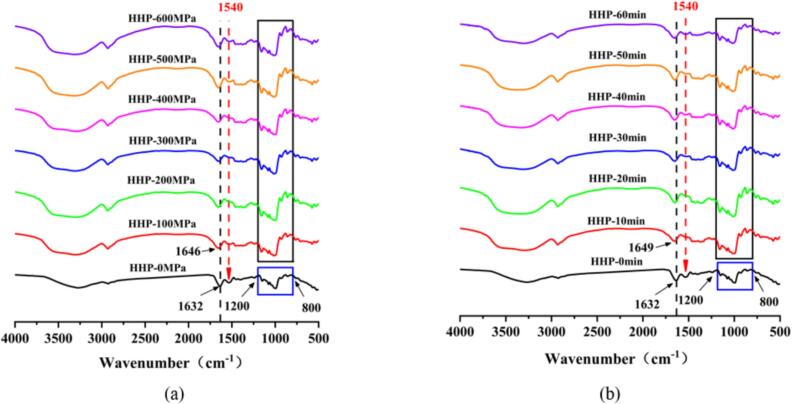
Infrared absorption spectra of lotus seed starch–protein blends undergoing HHP treatment with different treated conditions. (a) The HHP-0, 100, 200, 300, 400, 500, 600 MPa indicates lotus seed starch-protein blends with different treated pressure for 10 min. (b) The HHP-0, 10, 20, 30, 40, 50, 60 min indicates lotus seed starch–protein with different holding time for 400 MPa.

Infrared absorption spectra of the LS–LP blends under different HHP conditions are shown in Figs. 1a and b. According to the results, no new absorption peak appeared after subjecting samples to HHP treatments, suggesting that the treatment induces physical rather than chemical modifications (Guo, Liu, Zeng, & Zheng, 2013). HHP affects protein molecules by altering noncovalent bonds and subsequent intramolecular or intermolecular bond breaking caused by HHP treatment (Messens, Van Camp, & Huyghebaert, 1997). Therefore, distinct differences were observed in the characteristic protein absorption peaks in the infrared spectra between treated and untreated samples. The absorption peak of untreated LS–LP blends at approximately 1632 cm^−1^ (amide I band) that was blue-shifted to approximately 1646 and 1649 cm^−1^ after HHP treatment. The shift suggests that the spatial structure of proteins changed from the original stretching *β*-pleated sheet, antiparallel *β*-pleated sheet, and weak vibration of *β*-pleated sheet to the random coiled and *α*-helical structure with more intramolecular hydrogen bonds, thus maintain an orderly arrangement of protein molecules (Mejri, Rogé, BenSouissi, Michels, & Mathlouthi, 2005). The increase in *α*-helical content indicates increased hydrogen bonds in the system and improves hydration of proteins (Qamar, Bhandari, & Prakash, 2019). The observation revealed that hydrogen bonds in LS–LP blends increased under HHP treatment.

The absorption peak at approximately 1540 cm^−1^, which was attributed to the amide II band of the amino group (N—H) of LP, was observed in all spectra (Geng et al., 2022). The strength of this peak initially increased and then decreased with increasing HHP treatment levels. The decrease in peak strength could be attributed to the more evenly dispersed protein molecules interacting with starch molecules at HHP treatment lower than 400 MPa (Puppo et al., 2004; Qamar et al., 2019). However, the protein structure was flocculated under HHP conditions over 400 MPa to 600 MPa, which in turn, enhanced the interaction between protein intermolecular bonds and increased peak strength (Patel, Singh, Havea, Considine, & Creamer, 2005). No significant difference was observed in the peaks of LS–LP blends at approximately 1540 cm^−1^ with an increase in HHP treatment holding time (10–60 min) at the same pressure (400 MPa).

Changes in the starch structure, particularly the stretching vibrations of C—H, C—C, and C–OH, as well as the conformation and hydration of starch polymers are reflected in the infrared spectra range from 800 to 1200 cm^−1^. The untreated LS-LP blends exhibited relatively weak absorption peaks, which could be due to the addition of proteins with stable spatial structures that hinder the absorption peak vibration. The high absorption strength of peaks in the 800–1200 cm^−1^ range could be attributed to depolymerization of proteins and destruction of their stable structure under HHP treatment (Puppo et al., 2004). Conversely, starch structure was changed under HHP treatment, which increased the strength of the absorption peak vibration (Conde et al., 2022).

### 3.2 UV–vis spectroscopy

**Fig. 2:**
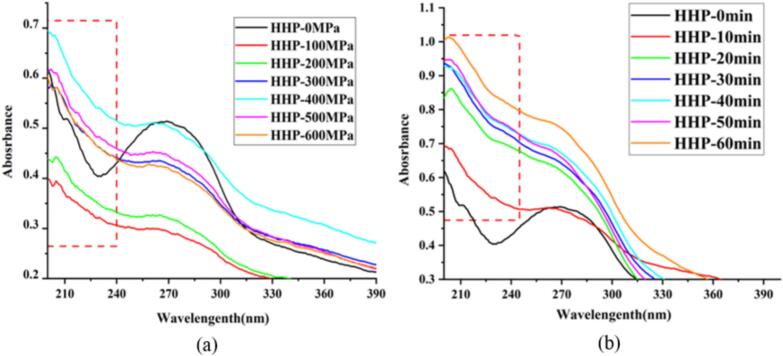
UV–visible absorption spectra of lotus seed starch–protein blends undergoing HHP treatment with different treated conditions. Note: (a) The HHP-0, 100, 200, 300, 400, 500, 600 MPa indicates lotus seed starch–protein with different treated pressure for 10 min. (b) The HHP-0, 10, 20, 30, 40, 50, 60 min indicates lotus seed starch–protein with different holding time for 400 MPa.

UV–Vis spectroscopy can effectively elucidate the formation of LS-LP blends (Guo et al., 2013). The absorption band associated with protein secondary structures, within the wavelength range of 200–240 nm (Guo et al., 2021). The absorption spectra of LS–LP blends (Figs. 2a) in the 200–240 nm range exhibited variations, with the peak at approximately 228 nm gradually diminishing. The UV–Vis spectra of LS–LP blends under 100–200 MPa exhibited low intensity curves at 200–240 nm, which is associated with high starch solubility and less influence on protein structures in LS–LP blends under HHP treatment. A previous study revealed that the protein structure was not destroyed under 100 MPa treatment with nearly content of sulfhydryl in untreated proteins, which are essential for protein aggregation (Chen et al., 2014). The intensities of the absorption spectra (Fig. 2a and b) of treated LS–LP blends under HHP treatment of 100–400 MPa increased with increasing pressure levels. This could be because of increased interaction between protein and starch molecules to form LS–LP blends at high pressure, leading to a loosening the protein structure and disrupting the arrangement of hydrogen bonds between starch molecules (Alvarez et al., 2008). However, above 400–600 MPa, the absorption spectra intensities of treated LS-LP blends decreased with further pressure increases. This decrease could be due to protein denaturation at high pressures, accompanied by flocculation, which enhances protein interactions and reduces starch interactions.

The effects of increasing holding times (from 0 to 60 min) at 400 MPa on the UV–Vis spectra of LS-LP blends are depicted in Fig. 2b. The UV–Vis spectral intensities of the treated LS–LP blends were greater than those of the untreated samples within the 200–240 nm range, suggesting that extended holding times can augment interactions between starch and protein molecules. This enhancement is likely due to the loosened protein structure interacting with the distorted arrangement of starch molecules with large particles (Conde et al., 2022; Guo et al., 2013).

### 3.3 SEM

**Fig. 3:**
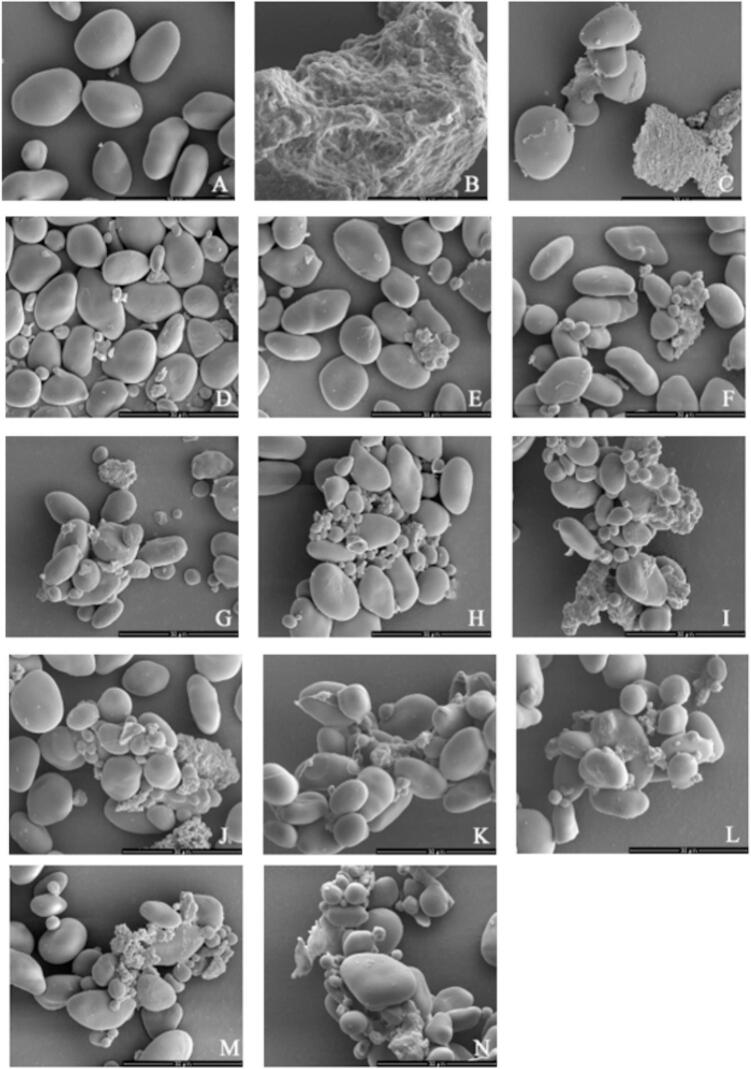
Scanning electron microscopy images (×5000) of lotus seed starch-protein blending system under different high hydrostatic pressure treated conditions. A: Lotus seed starch. B: Lotus seed protein. C: The untreated lotus seed starch-protein blending system. Lotus seed starch-lotus seed protein blending: D(100 MPa, 10 min), E(200 MPa, 10 min), F(300 MPa, 10 min), G(400 MPa, 10 min), H(500 MPa, 10 min), I(600 MPa, 10 min), J (400 MPa, 20 min) K(400 MPa, 30 min), L(400 MPa, 40 min), M(400 MPa, 50 min), N(400 MPa, 60 min).

The granule structures of LS–LP blends subjected to different HHP treatments were examined by SEM, depicted in Fig. 3. The lotus seed starch granules exhibited an oval shape with smooth surfaces (Fig. 3A). The microstructure of lotus seed protein powder with aggregated structure and untreated LS–LP blends, characterized by an aggregated structure, and the untreated LS-LP blends, which showed dispersed starch granules and protein aggregates, are presented in Fig. 3B and C, respectively. The surfaces of lotus seed starch granules were rough and had concave structures, while the lotus seed protein aggregates were dispersed at high pressure levels. The LS–LP blends treated with HHP of 100–200 MPa for 10 min, displayed lotus seed protein adsorbed onto the starch surface (Figs. 3D and E). More lotus seed starch granules were in contact with protein aggregates at high pressure levels. Furthermore, more spherical particles appeared (Figs. 3F–I), which resulted in the loosening of the lotus seed protein structure, in turn, enhancing interactions with lotus seed starch granules (Puppo et al., 2004). Under high pressure levels of 500–600 MPa, lotus seed proteins coagulated, leading to an increase in aggregation of lotus seed proteins as shown in Figs. 3H and I (Patel et al., 2005). The LS–LP blends treated with HHP of 400 MPa for 10–60 min are shown in Figs. 3G. J–N. Extended holding times promoted the denaturation of lotus seed proteins, exposing more binding sites for interaction with lotus seed starch. This could account for the increased absorption of lotus seed proteins around the starch granules, as shown in Figs. 3G. J–N (Lin & Fernández-Fraguas, 2020).

### 3.4 XRD

**Fig. 4:**
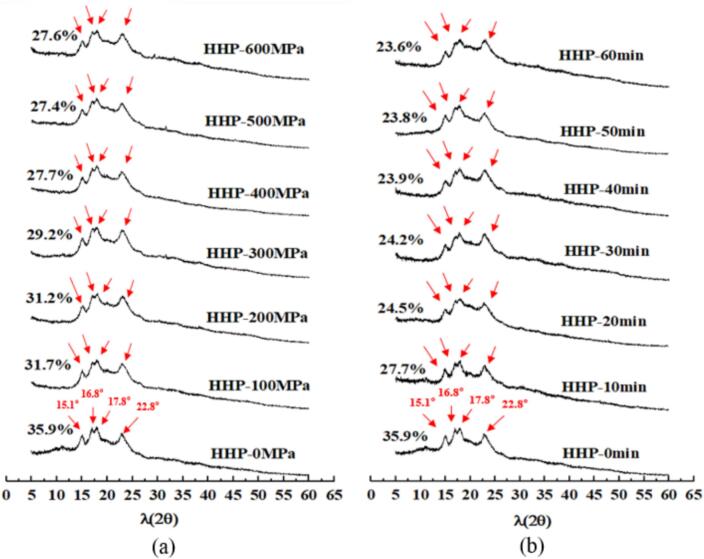
X-ray diffraction of lotus seed starch–protein blends undergoing HHP treatment with different treated conditions. (a) The HHP-0, 100, 200, 300, 400, 500, 600 MPa indicates lotus seed starch–protein with different treated pressure for 10 min. (b) The HHP-0, 10, 20, 30, 40, 50, 60 min indicates lotus seed starch–protein with different holding time for 400 MPa.

“A”, “B”, and “C” types are the three major XRD patterns and diffraction peaks observed for crystalline starch granules. Type “A” X-ray patterns exhibit four strong diffraction peaks at approximately 2θ = 15°, 17°, 18°, and 23°. Type “B” starch granules exhibited the most intense diffraction peak at 2θ = 17° and type “C” starch granules present a combination of “A” and “B” type patterns (Liu, Wu, Chen, & Chang, 2009). The XRD patterns of the LS–LP blends are shown in Fig. 4. Both untreated and treated LS-LP blends showed type “C” crystalline starch granules with prominent diffraction peaks at 2θ =15.1°, 16.8°, 17.8°, and 22.8°, and weak diffraction peaks at approximately 20° and 26°. Previous our studies indicate that HHP can induce a transition in the starch crystal structure from type “C” to type “B” under elevated processing pressures (Guo et al., 2015). Conversely, the incorporation of protein impedes this transformation, resulting in alterations solely to the starch's crystallinity. Changes in crystallinity can reflect the double helix structure of the remaining amylopectin in starch complexes (Oh, Pinder, Hemar, Anema, & Wong, 2008). As pressure increases and holding time during HHP treatment extends, there is a noticeable reduction in the crystallinity of LS-LP blends. This decrease may be attributed to the fact that the helical structures of certain starch molecules are released as a result of the HHP treatment. Such a release can lead to enhanced interactions between these starch molecules and other components, such as proteins and water molecules. Additionally, water molecules penetrate the starch granules during HHP treatment, causing the double helices in the crystalline region of starch to unwind and become more susceptible to disruption, thereby affecting the stability of the blends (Liu, Wang, Cao, Fan, & Wang, 2016).

### 3.5 Pasting properties of LS–LP blends

The pasting properties of LS–LP blends following HHP treatment, including the pasting temperature, peak viscosity, hold-through, final viscosity, breakdown, and setback are listed in Table 1. Except for setback, all pasting parameters increased at 400 MPa and then decreased with further increases in HHP intensity (Table 1(a)). Previous research indicates that gelatinization indicators in starch generally diminish under HHP treatment without protein addition (Liu, Wang, Cao, Fan, & Wang, 2016). In contrast, the pasting parameters of starch increase under HHP treatment after protein addition, which could be attributed to the enhancement of protein hydrophilicity by the HHP treatment (Wang et al., 2022). This accounts for the increased water absorption capacity and consequent viscosity rise in LS-LP blends as pressure increases from 0 to 400 MPa, which increased viscosity of the blends. This result is consistent with the findings of Eliasson (1983) who investigated the effect on starch gelatinization by adding protein, which could be due to the transfer of water from starch to protein. The increased breakdown and setback viscosities indicated that the pasting ability of LS–LP blends decreased with increasing pressure, which was caused by the distorted structures of starch and protein molecules under HHP treatment (Wang et al., 2022). However, blends treated at 500–600 MPa pressures showed a downward trend in peak, hold-through, final, and breakdown viscosities. This result may be due to the exposure of hydrophobic groups in proteins that subsequently affected the water absorption capacity of the LS–LP blends (Wang et al., 2008).

**Table 1:** Pasting properties of lotus seed starch-protein blends undergoing HHP treatment with different treated condition.

(a)					
Samples	Peak Viscosity	Hold-Through	Breakdown	Final Viscosity	Setback
HHP-0 MPa	491 ± 75^ab^	464 ± 74^ab^	26 ± 0.7^a^	638 ± 132^a^	174 ± 57^a^
HHP-100 MPa	539 ± 77^ab^	516 ± 96^ab^	23 ± 18^a^	704 ± 154^a^	188 ± 57^a^
HHP-200 MPa	586 ± 117^b^	536 ± 114^ab^	50 ± 2^a^	751 ± 206^a^	215 ± 91^a^
HHP-300 MPa	585 ± 26^b^	539 ± 47^ab^	46 ± 21^a^	742 ± 101^a^	203 ± 53^a^
HHP-400 MPa	636 ± 52^b^	575 ± 61^b^	60 ± 9^b^	809 ± 136^a^	234 ± 74^a^
HHP-500 MPa	625 ± 73^b^	588 ± 90^b^	37 ± 16^a^	809 ± 146^a^	221 ± 55^a^
HHP-600 MPa	395 ± 28^a^	350 ± 7^a^	45 ± 21^a^	568 ± 54^a^	218 ± 47^a^


(b)					
Samples	Peak Viscosity	Hold-Through	Breakdown	Final Viscosity	Setback
HHP-0 min	491 ± 75^a^	464 ± 74^a^	26 ± 1^a^	638 ± 132^a^	174 ± 57^a^
HHP-10 min	636 ± 52^b^	575 ± 61^b^	60 ± 9^b^	809 ± 136^ab^	234 ± 74^ab^
HHP-20 min	693 ± 4^bc^	670 ± 3^bc^	22 ± 7^a^	961 ± 5^bc^	290 ± 2^b^
HHP-30 min	701 ± 13^bc^	675 ± 14^bc^	26 ± 1^a^	972 ± 24b^bc^	296 ± 9^b^
HHP-40 min	694 ± 2^bc^	670 ± 2^bc^	24 ± 4^a^	959 ± 2^bc^	289 ± 0^b^
HHP-50 min	727 ± 16^bc^	705 ± 25^c^	22 ± 8^a^	1013 ± 45^c^	308 ± 19^b^
HHP-60 min	756 ± 51^c^	734 ± 50^c^	22 ± 8^a^	1047 ± 63^c^	312 ± 13^b^

Note: The HHP-0, 100, 200, 300, 400, 500, 600 MPa indicates lotus seed starch–protein blends with different treated pressure for 10 min. The HHP-0, 10, 20, 30, 40, 50, 60 min indicates lotus seed starch-protein with different holding time for 400 MPa. Data are mean ± standard deviation of duplicates. Values within a column followed by the different letter are significantly different (*p* > 0.05) as determined by Duncan test following ANOVA.

Apart from breakdown viscosity, all pasting parameters increased with HHP treatment at 400 MPa for 10–60 min (Table 1(b)). Extended HHP holding times can induce protein denaturation and expansion by exposing more hydrophilic groups, which enhances water absorption by the LS–LP blends to increase viscosity (Oh et al., 2008). Additionally, the viscosity of starch increases with prolonged high hydrostatic pressure (HHP) treatment. No significant changes in the breakdown viscosity were observed for LS–LP blends under extended HHP treatment, which can be attributed to enhanced interactions among starch, protein, and water molecules, as well as the relatively low availability of free starch, protein, and water molecules.

### 3.6 DSC

The thermal properties of the LS-LP blends were evaluated using differential scanning calorimetry (DSC). The resulting enthalpy change (ΔH) and gelatinization temperatures (i.e., the onset temperature (To), peak temperature (Tp), and final temperature (Tc)) are shown in Table 2. We found that compared to lotus seed starch, the addition of protein significantly increased To and Tp while decreasing ΔH. This indicates that the addition of protein affects the gelatinization properties of starch. And after being subjected to high hydrostatic pressure under different treatment conditions, ΔH was enhanced to varying degrees, and the gelatinization temperature was reduced. This suggests that thermal stability might be influenced by HHP treatment and starch-protein interactions. Elevating the pressure from 100 to 400 MPa for 10 min led to a reduction in the enthalpy of the LS–LP blends (Table 2(a)). The reduction in enthalpy may stem from the disruption of starch's crystalline structure (Castro, Alexandre, Saraiva, & Pintado, 2020). The decrease in enthalpy could have resulted from the broken crystalline structure of starch, increased interactions between starch and protein molecules, and high hydrophilicity of proteins, which reduced hydration of starch. The increase observed at pressures of 400–500 MPa could be associated with increased interactions between starch and water molecules when proteins aggregated. The increased involvement of starch leads to high enthalpy values which cause the trend differed from that with no additional starch under HHP treatment (Jagadeesan, Govindaraju, & Mazumder, 2020). A further enthalpy decrease at 600 MPa could be due to excessive pressure, which could be because of excessive pressure, thereby leading to complete gelatinization of some starch molecules. Proteins can inhibit complete gelatinization of starch, thereby reducing the enthalpy to a certain extent (Li et al., 2020).

**Table 2:** Thermal properties of lotus seed starch-protein blends undergoing HHP treatment with different treated condition.

(a)				
Samples	To(°C)	Tp(°C)	Tc(°C)	ΔH(J/g)
LS	72.7	77.6	84.5	16.09
HHP-0 MPa	81.2	84.9	89	4.906
HHP-100 MPa	74.4	78.6	84.8	6.558
HHP-200 MPa	75.7	79.1	83.6	6.437
HHP-300 MPa	75.8	79.6	84.2	6.006
HHP-400 MPa	75.6	79.5	84.1	6.687
HHP-500 MPa	75.8	79.4	84.1	6.856
HHP-600 MPa	76.1	79.4	83.9	6.022


(b)				
Samples	To(°C)	Tp(°C)	Tc(°C)	ΔH(J/g)
LS	72.7	77.6	84.5	16.09
HHP-0 min	75.6	79.5	84.1	4.906
HHP-10 min	73.3	76.7	81.1	6.687
HHP-20 min	73.2	77.1	81.0	6.672
HHP-30 min	73.1	76.8	80.9	6.663
HHP-40 min	73.0	77.0	80.8	6.642
HHP-50 min	72.9	77.1	80.7	6.632
HHP-60 min	75.6	79.5	84.1	6.61

Note: The LS indicates lotus seed starch without other treatment. The HHP-0, 100, 200, 300, 400, 500, 600 MPa indicates lotus seed starch–protein blends with different treated pressure for 10 min. The HHP-0, 10, 20, 30, 40, 50, 60 min indicates lotus seed starch-protein with different holding time for 400 MPa. To: onset temperature; Tp: peak temperature; Tc: cease temperature; ΔH: gelatinization enthalpy.

A decrease in enthalpy change (ΔH) and gelatinization temperatures was noted in LS–LP blends subjected to 400 MPa and holding time of 10–60 min (Table 2(b)). These findings imply that the crystalline structure of LS-LP blends undergoes continuous changes under the HHP condition of 400 MPa with extended holding times. The decrease in enthalpy change (ΔH) and gelatinization temperatures could be attributed to the destruction of the LS-LP blends' crystalline structure. Furthermore, the competition between starch molecules and expanded protein molecules with water molecules under HHP treatment with longer holding times can impede starch gelatinization, which in turn, decreases enthalpy and gelatinization temperatures.

### 3.7 Swelling power and solubility of LS–LP blends

**Fig. 5:**
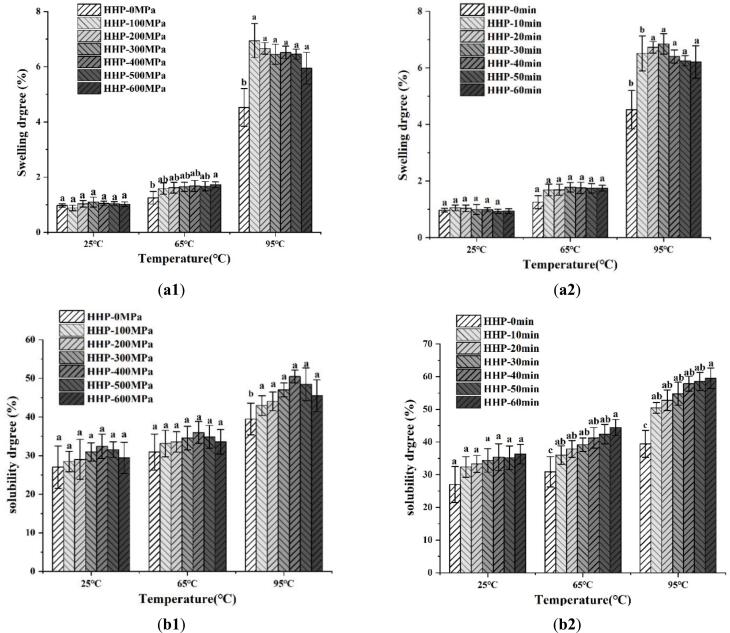
Swelling and Solubility Degree of lotus seed starch-protein blends undergoing HHP treatment with different treated pressure and holding time. The swelling (a1, a2) and solubility (b1, b2) degree of treated lotus seed starch-protein blends at three same temperatures (25 °C, 65 °C, 95 °C). The HHP-0, 100, 200, 300, 400, 500, 600 MPa indicates lotus seed starch–protein with different treated pressure for 10 min. The HHP-0, 10, 20, 30, 40, 50, 60 min indicates lotus seed starch-protein with different holding time for 400 MPa. Values within a column followed by the different letter are significantly different (*p* > 0.05) as determined by Duncan test following ANOVA).

The solubility and swelling degree of starch reflect the strength of interaction between starch and water molecules, as well as the interchain interaction strength within the amorphous and crystalline regions of starch (Lyon, Champagne, Vinyard, & Windham, 2000). Changes in the solubility and swelling degree of the LS–LP blends were determined as a function of temperature by heating at 25, 65, and 95 °C, corresponding to room temperature, gelatinization, and complete gelatinization temperatures, respectively (Fig. 5(a1–b2)). The swelling power and solubility values of the treated LS–LP blends were higher than those of untreated blends at all experimental temperatures, which could be attributed to the high hydrophilicity of the LS–LP blends and involvement of more molecules of the blends in hydration (Oh et al., 2008).

The swelling values of the LS–LP blends increased slightly increased with rising pressure and holding time when temperatures were maintained at 25 and 65 °C. In contrast, the results of LS–LP blends exposed to a high temperature (95 °C) contrasted those of LS–LP blends exposed to relatively low temperatures (25 and 65 °C). This discrepancy may arise from the unfolding of proteins at 100–600 MPa, which limited the absorption capacity of starch granules during heating by increasing interactions with starch molecules to inhibit water absorption and amylose leaching (Lyon et al., 2000). Moreover, the strong hydrogen bonds between starch and other molecules, along with competition between starch and protein molecules for water, can exacerbate the reduction in swelling (Zhao, Wang, Zheng, Chen, & Guo, 2019). The results are consistent with those of FTIR spectroscopy. The swelling power of the LS–LP blends continued to decrease significantly at a high pressure (600 MPa), which could have been caused by the complete gelatinization of starch and exposure of more hydrophobic groups in proteins (Leite, de Jesus, Schmiele, Tribst, & Cristianini, 2017; Wang et al., 2008).

The solubility values of the LS-LP blends under various HHP treatments and holding times are depicted in Fig. 5 (b1 and b2). The solubility values of original starch have been shown to decrease with an increase in HHP treatment. A similar trend was observed for LS–LP blends under pressure of 100–400 MPa. However, the LS–LP blends subjected to pressures of 500–600 MPa exhibited a contrasting trend. The results can be explained by additional proteins that provided more hydrophobic groups under high pressure, which reduce hydration (Alvarez et al., 2008). The continuous increase in solubility values of the LS–LP blends under high pressure for extended holding times could be attributed to the continuous exposure of hydrophilic groups in proteins, thereby enhancing solubility.

## 4 Conclusions

This study examined the effects of different HHP treatments and holding times on LS–LP blends. Under different HHP conditions, the secondary structure of proteins was depolymerized, and the conformation of starch molecules was modified, promoting interactions between starch and protein molecules. As a result, LS–LP blends with different particle, aggregate, and short-range structures, along with varied thermal and gelatinization properties were formed. The structures of the LS–LP blends under high and low pressures at the same holding time showed significant differences. Low pressures (0–400 MPa, 10 min) destroyed starch and protein structures and increased interactions between molecules, whereas high pressure caused protein aggregation and starch gelatinization, thereby reduce interactions between molecules, as evidenced by FTIR spectroscopy. UV–Vis spectroscopy revealed an increase in hydrogen bonds, which bolstered interactions between starch and protein molecules under HHP treatment, leading to the formation of new stable structures. SEM observations indicated that protein aggregates were adsorbed onto the surfaces of starch molecules. The treated LS-LP blends exhibited the same C-type crystalline structure, with crystallinity reduced to varying degrees due to the effects of HHP treatment, suggesting damage to the starch's crystalline structure. Additionally, the hydrophilic groups in proteins increased under HHP treatment, enhancing the water absorption capacity, solubility, swelling power, and viscosity of the LS-LP blends. The improved properties of the LS-LP blends, as a result of the HHP treatment, not only affect their structural integrity and physicochemical behavior but also significantly expand their industrial potential. The findings of this study have deepened our understanding of how these changes impact the structure and physicochemical properties of LS-LP blends under various HHP conditions, thereby providing a theoretical foundation for the development of advanced processing techniques for lotus seeds. Furthermore, this research introduces a novel, no-thermal method for preparing LS-LP blends.

## Author contribution

The manuscript was written through contributions of all authors. All authors have given approval to the final version of the manuscript.

## CRediT authorship contribution statement

**Sidi Liu:** Writing – review & editing, Writing – original draft, Validation, Methodology, Formal analysis, Conceptualization. **Ru Jia:** Writing – review & editing, Methodology, Investigation. **Wenjing Chen:** Methodology, Data curation. **Wenyu Chen:** Writing – review & editing, Resources. **Baodong Zheng:** Writing – review & editing, Supervision. **Zebin Guo:** Writing – review & editing, Supervision, Resources, Funding acquisition, Conceptualization.

## Declaration of competing interest

The authors declare that they have no known competing financial interests or personal relationships that could have appeared to influence the work reported in this paper.

## Data Availability

Data will be made available on request.
